# "One Medicine" for Animal and Human Health

**DOI:** 10.3201/eid1012.AC1012

**Published:** 2004-12

**Authors:** Polyxeni Potter

**Affiliations:** * Centers for Disease Control and Prevention, Atlanta, Georgia, USA

**Keywords:** Art and science, emerging infectious diseases, Zoonoses, Edward Hicks, Peaceable Kingdom, cover text, West Nile virus, avian influenza

**Figure:**
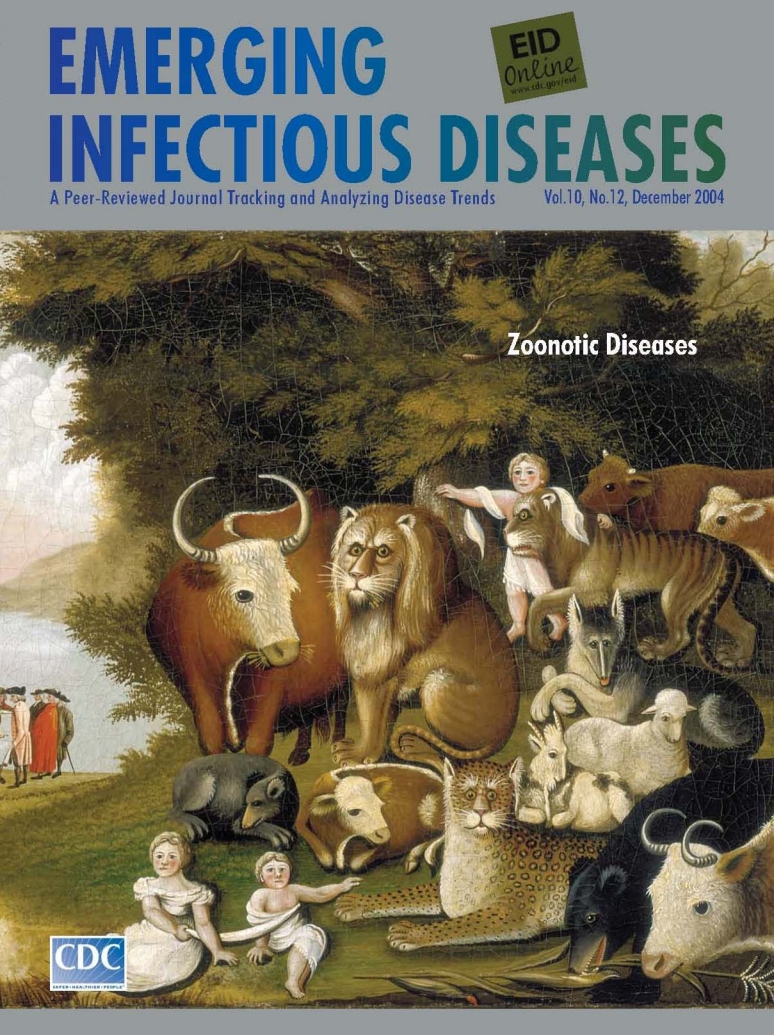
**Edward Hicks (1780–1849). Peaceable Kingdom (c. 1833).** Oil on canvas (44.5 cm x 60.2 cm). Worcester Art Museum

"The wolf also shall dwell with the lamb, and the leopard shall lie down with the kid, the calf, and the young lion, and the fatling together, and a little child shall lead them." These biblical lines (Isaiah 11:6–9) provided Edward Hicks the allegorical framework in which to approach a theme of special fascination to him: the peaceable kingdom, an idyllic world in which all creatures live in harmony. He painted more than 100 versions of this theme, 62 of which have survived (*1*).

A native of Bucks County, Pennsylvania, Hicks was orphaned in infancy and raised by a Quaker family. At 13 years of age, he was apprenticed to a carriage maker and learned to decorate coaches. He showed natural talent for decorative painting and, even without academic training, became very successful. "I am now employing four hands, besides myself, in coach, sign and ornamental painting, and still more in repairing and finishing carriages," he wrote reflecting on his experiences, "and I think I should find no difficulty in doubling my business" (*2*).

As a young man, Hicks set out to explore the wild side of life but soon returned to the Quakers and became a popular itinerant preacher. This Religious Society of Friends espoused the principles of equality and nonviolence but frowned upon artistic ventures as too worldly. Hicks abandoned his ornamental painting business to become a farmer only to relent, reluctantly, in middle age and move from commercial decoration to easel painting. Inspiration came mostly from his religious faith and missionary work among Native American tribes (*3*).

Born into a new nation, just 4 years after the Declaration of Independence, Hicks became part of an 18th- and 19th-century American tradition that produced provincial or folk art (portraits, landscapes, religious, and historical themes) characterized by a naïve style. This style, the hallmark of self-taught artists who arrived at their art through a journey of discovery and realization of innate talent, often flourished in rural areas and small towns (*4*). Outside traditional rules of perspective and proportion, naïve style relied on intuitive organization and structure, and was imaginative, creative, and direct.

Even though in his day he gained notoriety as an impassioned preacher, Hicks is now remembered for his art. His paintings, a continuation of his religious beliefs, explored ethical and spiritual dilemmas and commemorated historical events. Human figures, animals, and landscapes were created to embody Quaker ideals. Color, size, proportion, placement, and other elements were used as symbols to compose a moral message, which was often inscribed on the frame.

In a world marred by strife—between nations, between animals and humans, between animals themselves—Hicks tapped into the universal wish for harmony and peace. Well ahead of his time, he invited to his kingdoms not only leading human figures and innocent children but also a consortium of animals whose presence he found indispensable. Domesticated animals, part of his life as a farmer, appeared in realistic detail, but wild beasts were more idealized and decorative.

In The Peaceable Kingdom on this month's cover of Emerging Infectious Diseases, Hicks once more assembled the world's creatures for an idyllic group portrait. Against a lighted backdrop of trees and river banks, animals and children gathered in the foreground. In mid-panel, leading Quaker William Penn concluded a peace treaty with the Lenni Lenape tribe. The colors were solid, the light well focused, and the curves of animal frames and horns gracefully outlined. Yet, Hicks was not denying tensions in the universe.

The animals, whose anthropomorphic features betrayed human emotions, seemed puzzled and apprehensive. Even as the bull offered the lion hay, the king of beasts seemed stiff and uneasy. Even as the lamb cuddled up, the wolf wore a noncommittal glare. The world's creatures may have been tamed, but peace in the scene seems precarious.

The connectivity that Hicks sensed between humans, animals, and the universe was greater than the artist could have imagined. The intensity in the animals' eyes was not the only troubling element in the picture. In the dander and under their breath, in the soil and in the water, on the leaves and the clothing of the dignitaries, lay creatures unknown to Hicks, microorganisms, insidiously moving from animals to humans, eating, multiplying, sharing, spreading, connecting. Even if Hicks could have arranged a perfectly peaceable kingdom, strife would have continued beneath the surface through the transmission of disease.

Not long after Hicks' death in 1849, German pathologist Rudolf Virchow (1821–1902) coined the term zoonosis, verifying the essential link between animal and human health (*5*). This link, further complicated by the emerging nature of disease and the ethical, ecologic, social, and economic values placed on the relation between humans and their pets, livestock, or fellow inhabitants of nature, has not been uniformly acknowledged or exploited—even in the face of AIDS, Ebola, West Nile virus, avian influenza, bovine spongiform encephalopathy, and SARS.

In the 1980s, American epidemiologist Calvin Schwabe proposed a unified human and veterinary approach against zoonotic diseases. This approach, "one medicine" (*6*), upholds Virchow's principles and affirms Hicks' wish for the control of subversive elements, whether they interfere with harmonious animal and human interaction or they disrupt animal and human health.
